# Corylin increases the sensitivity of hepatocellular carcinoma cells to chemotherapy through long noncoding RNA RAD51-AS1-mediated inhibition of DNA repair

**DOI:** 10.1038/s41419-018-0575-0

**Published:** 2018-05-10

**Authors:** Chin-Chuan Chen, Chi-Yuan Chen, Shir-Hwa Ueng, Chuen Hsueh, Chau-Ting Yeh, Jar-Yi Ho, Li-Fang Chou, Tong-Hong Wang

**Affiliations:** 1 Tissue Bank, Chang Gung Memorial Hospital, Tao-Yuan, 33305 Taiwan; 2 Graduate Institute of Natural Products, Chang Gung University, Tao-Yuan, 33303 Taiwan; 3 Graduate Institute of Health Industry Technology and Research Center for Industry of Human Ecology, College of Human Ecology, Chang Gung University of Science and Technology, Tao-Yuan, 33303 Taiwan; 4 Department of Anatomic Pathology, Chang Gung Memorial Hospital, Chang Gung University School of Medicine, Tao-Yuan, 33305 Taiwan; 5 Liver Research Center, Department of Hepato-Gastroenterology, Chang Gung Memorial Hospital, Tao-Yuan, 33305 Taiwan; 6 Department of Pathology, and Graduate Institute of Pathology and Parasitology, Tri-Service General Hospital, National Defense Medical Center, Taipei, 11490 Taiwan; 7 Kidney Research Center, Chang Gung Memorial Hospital, Tao-Yuan, 33305 Taiwan

## Abstract

Corylin, a biologically active agent extracted from *Psoralea corylifolia* L. (Fabaceae), promotes bone differentiation and inhibits inflammation. Currently, few reports have addressed the biological functions that are regulated by corylin, and to date, no studies have investigated its antitumor activity. In this study, we used cell functional assays to analyze the antitumor activity of corylin in hepatocellular carcinoma (HCC). Furthermore, whole-transcriptome assays were performed to identify the downstream genes that were regulated by corylin, and gain-of-function and loss-of-function experiments were conducted to examine the regulatory roles of the above genes. We found that corylin significantly inhibited the proliferation, migration, and invasion of HCC cells and increased the toxic effects of chemotherapeutic agents against HCC cells. These properties were due to the induction of a long noncoding RNA, RAD51-AS1, which bound to RAD51 mRNA, thereby inhibiting RAD51 protein expression, thus inhibiting the DNA damage repair ability of HCC cells. Animal experiments also showed that a combination treatment with corylin significantly increased the inhibitory effects of the chemotherapeutic agent etoposide (VP16) on tumor growth. These findings indicate that corylin has strong potential as an adjuvant drug in HCC treatment and that corylin can strengthen the therapeutic efficacy of chemotherapy and radiotherapy.

## Introduction

Hepatocellular carcinoma (HCC) is the most common liver cancer and is the fifth most prevalent cancer globally^1^. Every year, ~700,000 people worldwide receive a diagnosis of HCC^2^. Currently, the mainstay treatment of HCC is surgical resection, and patients with late-stage cancer and distal metastases receive chemotherapy^3, 4^. The latter can result in DNA damage in cancer cells and induce apoptosis. Nevertheless, mutations in the DNA repair systems of many HCC cells result in excessive activation and poor chemotherapeutic efficacy^5, 6^. Therefore, in clinical practice, chemotherapy is often combined with DNA repair inhibitors to increase its therapeutic efficacy^7–10^.

The use of traditional Chinese medicine (TCM) in the treatment of diseases has a long history in China^11–13^. Compared with Western medicine, TCM provides another effective treatment option with relatively mild side effects^14–16^. In recent years, relevant studies on the use of TCM in cancer treatment have gradually attracted attention. Nevertheless, differences in the quality of TCM are caused by differences in the concentrations of natural biologically active ingredients in herbs and other substances, and this situation usually results in unstable therapeutic efficacy and limits the applications of TCM^17–19^. Therefore, to enhance and stabilize the therapeutic efficacy of TCM, many studies have focused on identifying and purifying the biologically active ingredients of the medicinal substances used in TCM^18^. With developments in chemical purification technologies and mass spectrometry, the active ingredients of many TCM substances have been successively purified, and their functions have been determined^20–23^. These extracts can be used at lower doses and can provide more specific therapeutic efficacies. Lately, many TCM-derived compounds, such as *trans*-resveratrol, artemisinin, and curcumin, have been tested in cancer treatment and have shown good efficacy^24–28^. Some of these drugs can significantly inhibit the growth and metastasis of HCC and significantly increase the survival times of patients^29–32^. In addition, many TCM extracts, such as *trans*-resveratrol, have the capacity to inhibit DNA damage repair, and these drugs can synergize with chemotherapy and radiotherapy to enhance their therapeutic efficacies^33, 34^.

*Psoralea corylifolia* L. (Fabaceae) is an herb that is commonly used in TCM in Asian countries and has antioxidant, anti-inflammatory, and anticancer effects^35–38^. This herb is often used in the treatment of inflammation due to bacterial infections. Corylin is a flavonoid compound that is extracted from *P. corylifolia* L., and the current understanding of its effects is limited. Corylin is known to promote bone differentiation and to inhibit inflammation by suppressing inducible nitric oxide synthase (iNOS) and cyclooxygenase (COX) expression that is induced by bacterial infection^39–41^. To date, no studies have examined the anticancer effects of corylin. In this study, we used cell and animal models to analyze the antitumor activity of corylin in HCC and to elucidate its molecular mechanisms of action. We found that corylin can inhibit DNA repair in HCC cells. This action is due to the induction of a long noncoding RNA (lncRNA) called RAD51-AS1, which binds to RAD51 mRNA and downregulates the RAD51 protein. This change increases the sensitivity of HCC cells to chemotherapy and radiotherapy.

## Results

### Corylin inhibits the proliferation, migration, and invasion capacity of HCC cells

**Fig. 1:**
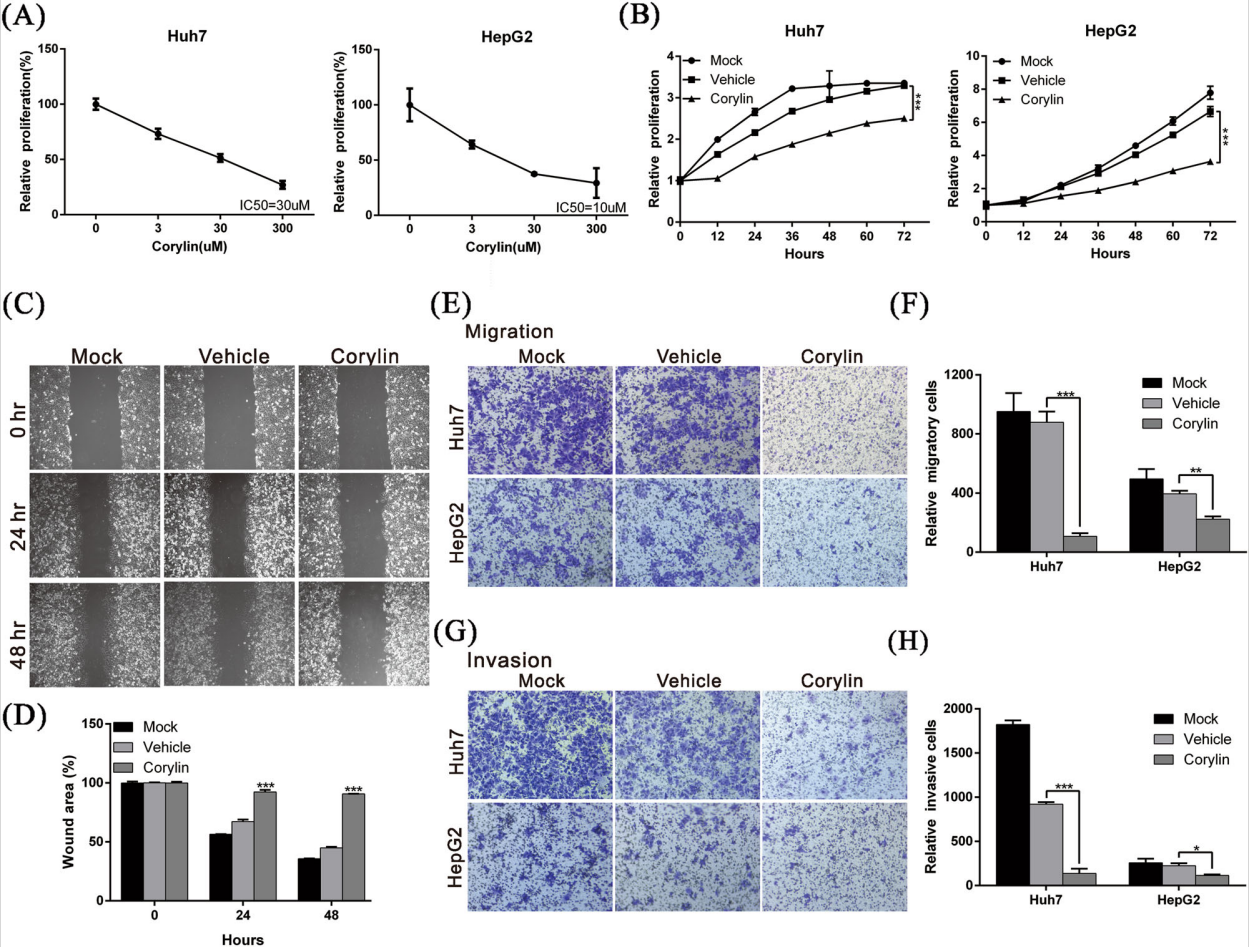
Corylin inhibited the proliferation, migration, and invasion capacities of HCC cells. **a** Sensitivity of HCC cell lines to corylin treatment. Cells were treated with different concentrations of corylin for 72 h. Viability of the treated cells were assayed using an xCELLigence real-time cell analyzer. The inhibitory concentration (IC_50_) of corylin on HCC cells was calculated by Graphpad prism 6 software. **b** Huh7 and HepG2 cells were treated with 30 μM corylin, and the cell proliferation capacities were monitored at the indicated time points using an xCELLigence real-time cell analyzer. ***p < 0.001, as assessed using the Student’s *t-*test. **c** Huh7 cells were untreated or treated with 30 μM corylin, and the wound-healing abilities of cells were monitored at the indicated time points. The quantification of the cell wound-healing assay is presented in **d**. ***p < 0.001. **e** Cell migration capacities of Huh7 and HepG2 cells with/without 30 μM corylin treatment were compared using transwell assays. The quantitative cell migration assay results are shown in **f**. **p < 0.01, ***p < 0.001. **g** Invasion assays were performed using matrigel-coated polyethylene terephthalate membrane inserts. Five different 200 × fields were imaged to quantify the numbers of migrating or invading cells. The quantitative cell invasion assay results are shown in **h**. ***p < 0.001. All data were expressed as the mean ± S.D. of three independent experiments

To determine whether corylin has therapeutic effects on HCC, we used different concentrations of corylin to treat HCC cell lines HepG2 and Huh7. We observed inhibitory effects on cell proliferation starting at a concentration of 3 μM for 72 h treatment, and these effects were dose dependent. The half-maximal inhibitory concentration (IC_50_) of corylin toward HepG2 and Huh7 cells was 10 and 30 μM, respectively (Fig. 1a). The results showed that when 30 μM corylin was incubated with HepG2 or Huh7 HCC cells, cell proliferation was significantly inhibited. Compared with the control group that was treated with vehicle (dimethyl sulfoxide, DMSO), the growth rates of the HepG2 and Huh7 cells that were treated with corylin for 72 h showed 45.3% and 23.9% inhibition (Fig. 1b), respectively. The above results indicated that corylin inhibited the growth of HCC cells.

Cancer cell metastasis and invasion are among the main causes of cancer resistance to treatment. To determine whether corylin affected the metastasis and invasion capabilities of HCC cells, we used wound-healing and transwell assays to analyze the effects of corylin on cell migration and invasion. The results of the experiments revealed that 30 μM corylin significantly inhibited the cell migration capacities of both cell lines (Fig. 1c–f). The invasion assay also showed that corylin could suppress the invasiveness of HepG2 and Huh7 cells by 49.3% and 85%, respectively (Fig. 1g, h).

### Corylin increases the sensitivity of HCC cells to chemotherapy and radiotherapy

**Fig. 2:**
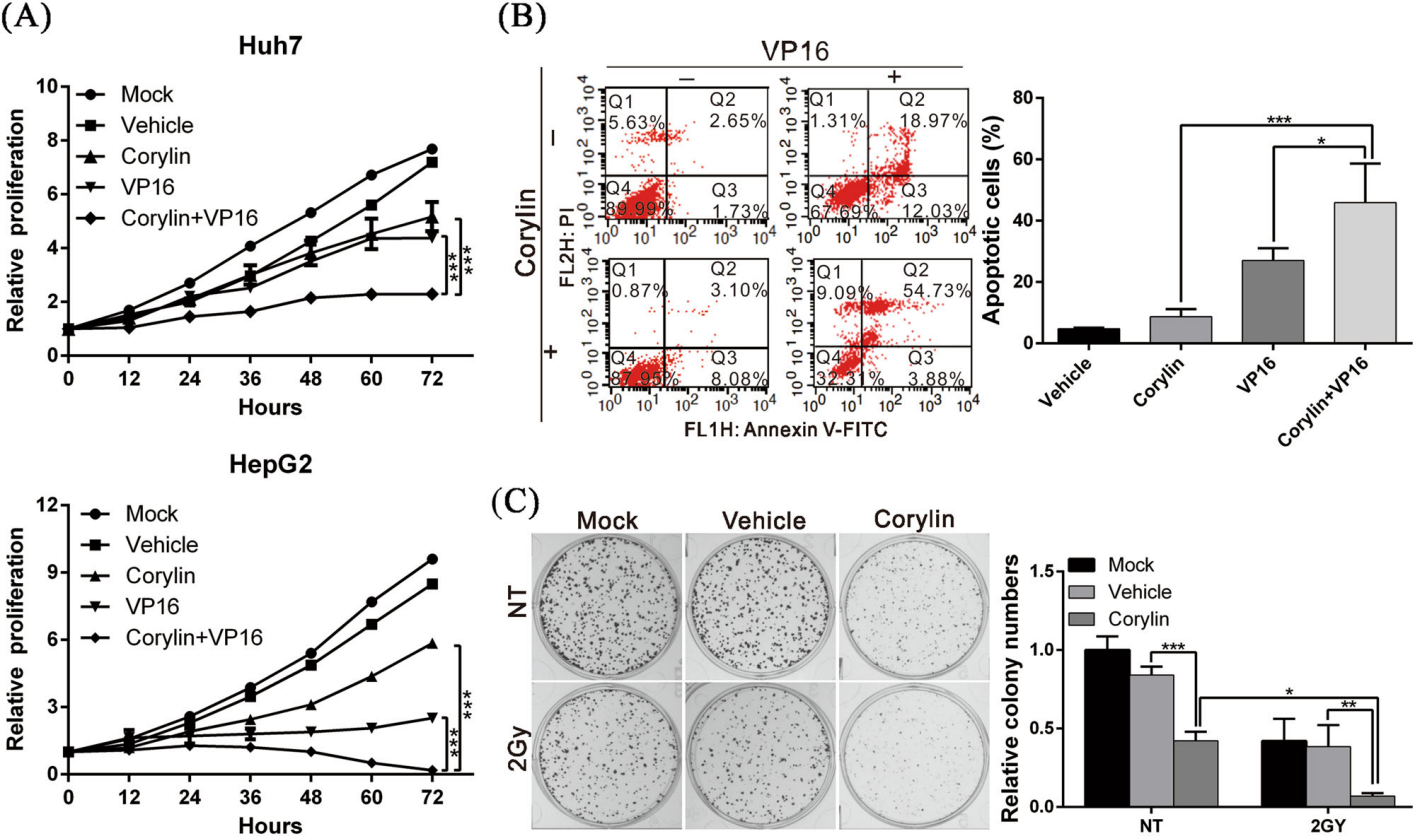
Corylin increased the sensitivity of HCC cells to chemotherapy and radiotherapy. **a** The cell proliferation capacities of Huh7 (upper panel) and HepG2 (lower panel) cells with different treatments were monitored using an xCELLigence real-time cell analyzer. Data were expressed as the mean ± S.D. of three independent experiments. **b** Flow cytometry analysis of cell apoptosis. Huh7 cells were treated with 30 μM corylin in the presence or absence of 200 μM etoposide (VP16) for 48 h, and cell apoptosis was determined by flow cytometry after Annexin V/PI staining (left panel). The quantification of the apoptotic cells is presented in the right panel. ***p < 0.001. **c** Effect of corylin on the radiosensitivity of HCC cells. Huh7 cells were irradiated with Cs-137 at a dose of 2 Gy, and were then left untreated or treated with 30 μM corylin for 24 h. The cells were then cultured for an additional 10 days in the absence of any drug (left panel). The number of foci was scored, and the results are presented in the right panel. ***p < 0.001. All experiments were performed in triplicate

To elucidate the therapeutic efficacy of corylin when it is combined with another anticancer treatment, we combined corylin with etoposide (VP16), a chemotherapeutic agent, for a simultaneous treatment of HCC cells and analyzed the inhibitory effects of this combination on the growth of these cells. We found that corylin significantly increased the anticancer effects of etoposide (VP16). Additionally, the combination of the two drugs inhibited cancer cell growth by approximately onefold compared with the inhibition by either etoposide (VP16) or corylin alone (Fig. 2a). In addition, when flow cytometry was used to analyze the cellular apoptosis status, the results showed that the combination treatment had a significantly higher cytotoxic effect on the cancer cells than etoposide (VP16) alone and that a twofold increase (59% vs 31%) in the cellular apoptosis rate was evident (Fig. 2b). Furthermore, when corylin was combined with the irradiation of HCC cells, colony formation inhibition significantly increased compared with radiation alone (Fig. 2c). This finding revealed that corylin could sensitize HCC cells to chemotherapy or radiotherapy.

### Corylin decreases the DNA damage repair capacity of HCC cells by suppressing RAD51 expression

**Fig. 3:**
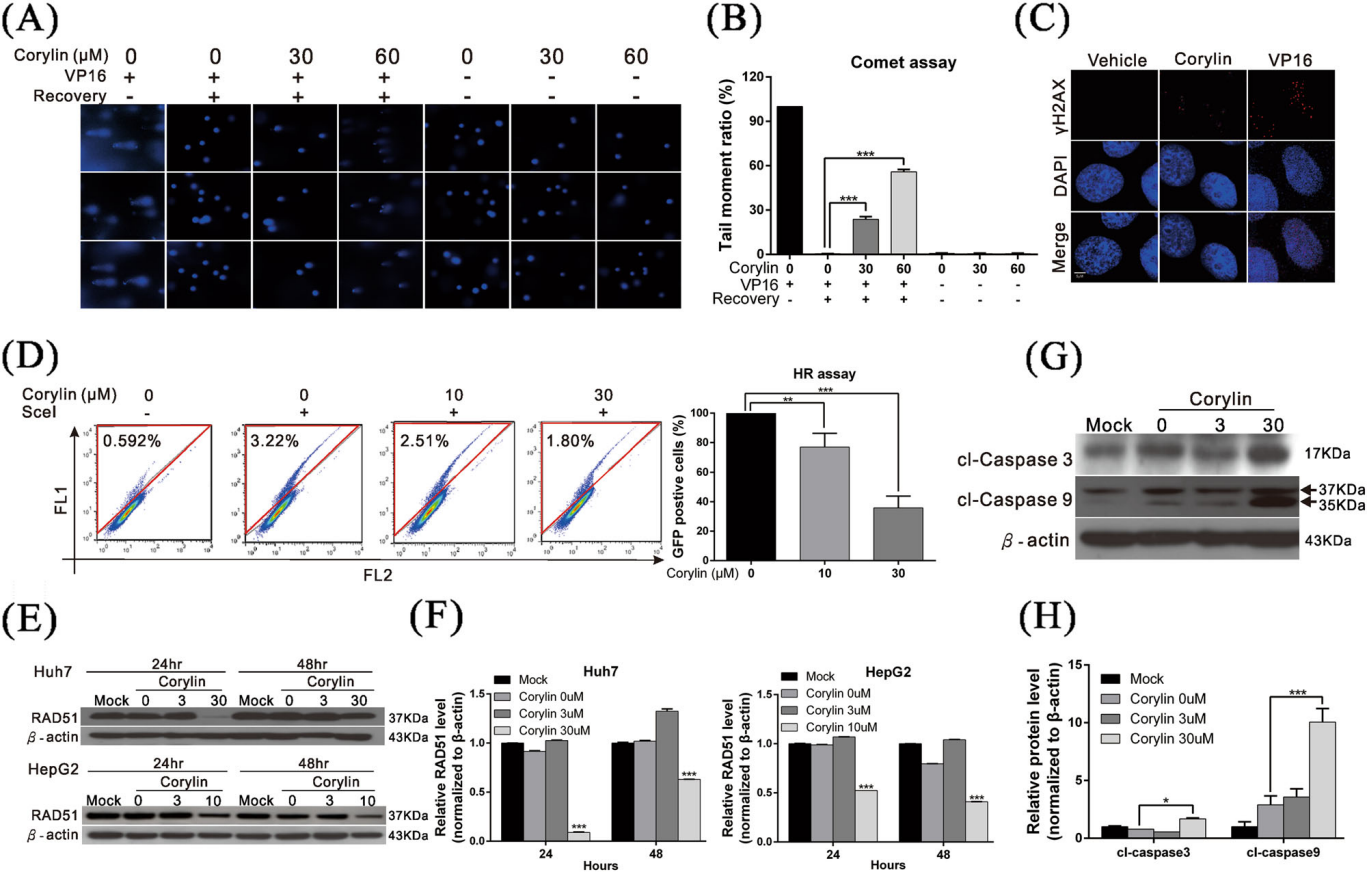
Corylin decreased the DNA damage repair capacity of HCC cells by suppressing RAD51 expression. **a** Comet assays represented the activity of DNA damage repair after treatment with different concentrations of corylin. Huh7 cells were treated with 200 µM etoposide (VP16) for 1 h, followed by treatment with different concentrations of corylin in etoposide (VP16)-free medium for 4 h. The cells were harvested and subjected to comet assays to detect the DNA repair activity. The quantitative DNA damage repair activity results are shown in **b**. **p < 0.01, ***p < 0.001. **c** Huh7 cells were treated with 30 µM corylin or 200 µM etoposide (VP16) for 24 h. Immunofluorescence analysis with the anti-rH2A antibody shows that etoposide (VP16) induces DNA breaks. **d** The results of flow cytometry show the effect of corylin on homologous recombination (HR) activity in Huh7 cells (left panel). Huh7 cells were transfected with both the DR-GFP vector and I-SceI expression vector with or without corylin treatment for 48 h to measure the HR-mediated repair capacity. The GFP-positive cells (red label) were then detected by BD FACSCalibur. The quantitative results are shown in the right panel. **e** Huh7 and HepG2 cells were treated with different concentrations of corylin for 24 h or 48 h, and cell lysates of treated cells were assayed for RAD51 expression level by western blotting. β-Actin served as an internal control. The quantitative results are shown in **f**. ***p < 0.001. **g** Huh7 cells were treated with different concentrations of corylin for 48 h, and then cell lysates were subjected to western blotting to detect the level of cleaved caspases 3 and 9. β-Actin served as an internal control. The quantitative results are shown in **h**. All data were expressed as the mean ± S.D. of three independent experiments. *p < 0.05, ***p < 0.001. cl-caspase 3/9 cleaved caspase 3/9

The above results showed that in addition to inhibiting the proliferation, migration, and invasion capacity of HCC cells, corylin increased the toxicity of radiotherapy and chemotherapy in HCC cells. The main mechanisms of action of radiotherapy and chemotherapy are the induction of cellular DNA damage, which results in apoptosis. To determine whether corylin participated in the regulation of DNA damage repair thereby increasing the sensitivity of HCC cells to chemotherapy and radiotherapy, the Huh7 cells were pre-treated with etoposide (VP16) to induce DNA breaks. After treatment with etoposide (VP16), cells were treated with different concentrations of corylin and the DNA damage repair capacity of cells was analyzed by the comet assay. We found that the DNA damage repair velocity of the cells treated with corylin was significantly slower than that of the cells in the control group (vehicle only), and this effect was dose dependent, indicating that corylin could inhibit the DNA damage repair capacity of cells (Fig. 3a, b). The immunofluorescence analysis showed that after the cells were treated with etoposide (VP16), the ratio of histone H_2_AX phosphorylation (*r*H_2_AX) increased, confirming that DNA breaks were induced by etoposide (VP16) (Fig. 3c).

Homologous recombination (HR) is the main mechanism by which cells repair a double-stranded DNA break. To determine whether corylin regulated HR, we used an HR reporter assay. The results showed that compared with the control group, corylin significantly inhibited HR (Fig. 3d). Next, to elucidate the mechanisms by which corylin inhibited HR, we performed western blotting to analyze the expression of RAD51 (one of the key proteins participating in HR). We found that after the cells were treated with corylin, RAD51 expression significantly decreased (Fig. 3e, f). In addition, we noticed that corylin significantly induced the expression and activation of caspases 3 and 9 in HCC cells (Fig. 3g, h) and promoted apoptosis. The above results revealed that corylin could inhibit DNA repair in HCC cells by inhibiting RAD51 expression, thereby strengthening the cytotoxic effects of chemotherapy and radiotherapy.

### Corylin can induce the expression of lncRNA RAD51-AS1 to downregulate the RAD51 protein

**Fig. 4:**
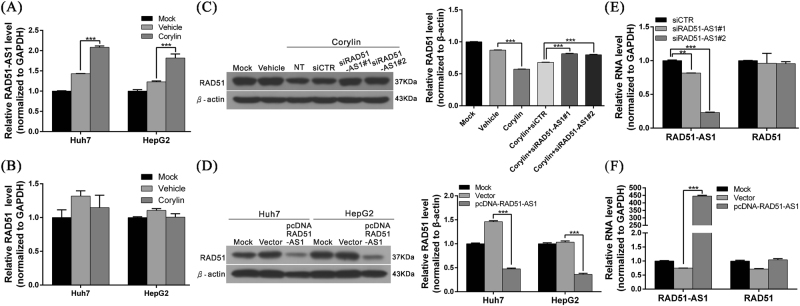
Corylin induced the expression of lncRNA RAD51-AS1 to downregulate the RAD51 protein. **a**, **b** Huh7 and HepG2 cells were treated with 30 μM corylin for 48 h, and the expression of RAD51-AS1 and RAD51 was analyzed by quantitative real-time RT-PCR. GAPDH served as an internal control. ***p < 0.001. **c** Huh7 cells were transfected with siRNA against lncRNA RAD51-AS1 (siRAD51-AS1) or non-targeting siRNA (si-CTR) at 50 nM, and then co-treated with corylin (30 μM). After 48-h treatment, cells were harvested and lysates were subjected to western blotting to detect the level of RAD51. β-Actin served as an internal control (left panel). The quantitative results are shown in the right panel. ***p < 0.001. **d** Huh7 and HepG2 cells were transfected with 1 μg pcDNA-RAD51-AS1 expressing plasmid or empty vector for 48 h. The protein level of RAD51 was analyzed by western blotting (left panel). The quantitative results are shown in the right panel. ***p < 0.001. **e**, **f** Real-time PCR analysis shows the mRNA levels of RAD51-AS1 and RAD51 during the above-mentioned two treatments. GAPDH served as an internal control. All data were expressed as the mean ± S.D. of three independent experiments. **p < 0.01, ***p < 0.001

Multiple studies have confirmed that lncRNAs have different mechanisms for the regulation of gene expression. To determine whether lncRNAs participate in the corylin-mediated RAD51 regulation, we employed whole-transcriptome sequencing to analyze the lncRNA expression statuses of corylin-treated Huh7 and HepG2 cells^42^. We found that the expression of a recently discovered lncRNA, RAD51-AS1, was significantly increased after corylin treatment. Afterward, we confirmed this finding by real-time PCR and verified that lncRNA RAD51-AS1 expression in corylin-treated Huh7 and HepG2 cells increased by 45.6% and 47.9%, respectively, compared with the control group (vehicle only; Fig. 4a). This result showed that corylin could induce the expression of lncRNA RAD51-AS1. Conversely, the above treatment did not have any effects on the RAD51 mRNA levels (Fig. 4b).

Several studies have confirmed that multiple antisense lncRNAs can form a duplex RNA structure with the sense strand (mRNA) and that this phenomenon can (1) stabilize the mRNA structure and increase protein expression, (2) induce RNA interference mechanisms that degrade double-stranded RNA, or (3) interfere with the binding of a ribosome to RNA and inhibit protein synthesis. To understand the regulatory mechanisms of lncRNA RAD51-AS1 affecting RAD51 mRNA, we applied small interfering RNA (siRNA) to silence the expression of RAD51-AS1 and performed real-time PCR and western blotting to analyze the mRNA and protein expression statuses of RAD51. The results showed that after the knockdown of RAD51-AS1, the RAD51 protein levels significantly increased. On the other hand, after the overexpression of lncRNA RAD51-AS1, the RAD51 protein levels significantly decreased (Fig. 4c, d). Nevertheless, during the abovementioned two treatments, the RAD51 mRNA levels did not significantly change (Fig. 4e, f). These results indicated that RAD51-AS1 could suppress RAD51 protein expression. This effect is likely mediated by the inhibition of RAD51 protein synthesis and not by RAD51 mRNA degradation via the induction of RNA interference.

### Corylin increases the sensitivity of HCC cells to a chemotherapeutic agent by inducing lncRNA RAD51-AS1

**Fig. 5:**
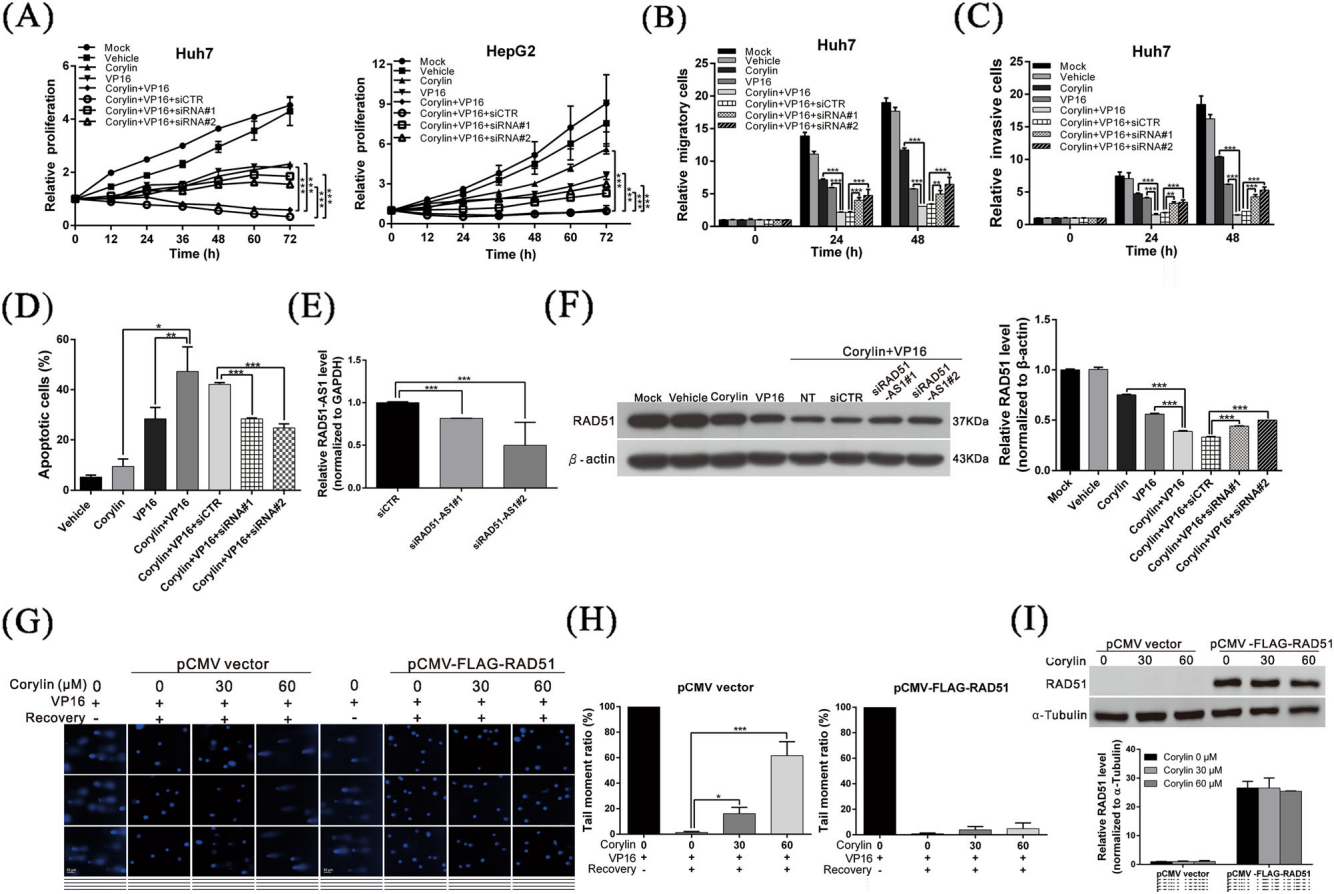
Corylin increases the sensitivity of HCC cells to a chemotherapeutic agent by inducing lncRNA RAD51-AS1. **a**–**d** The effects of corylin (30 μM) combined with etoposide (VP16) (200 μM) on cell proliferation, migration, invasion, and apoptosis with/without treatment with RAD51-AS1 siRNA (50 nM) in HCC cells. *p < 0.05, **p < 0.01, ***p < 0.001, as assessed using the Student’s *t-*test. **e** Real-time PCR analysis shows the mRNA levels of RAD51-AS1 during the siRNA treatments. **f** Western blot analysis shows the effect of the above treatments on the expression of RAD51 (left panel). The quantitative results are shown in the right panel. ***p < 0.001. **g** Comet assays show that the overexpression of the RAD51 protein attenuates the inhibition of DNA repair by corylin. The quantitative cell repair activity results are shown in **h**. All data were expressed as the mean ± S.D. of three independent experiments. *p < 0.05, ***p < 0.001. **i** Western blot analysis shows the effect of the above treatments on the expression of RAD51 (upper panel). The quantitative results are shown in the lower panel. ***p < 0.001

To verify whether the anticancer effects of corylin were mediated by lncRNA RAD51-AS1, we carried out a rescue assay. The results of the experiment revealed that after the cells were treated with both corylin and etoposide (VP16), the proliferation, migration, and invasion capacities of the cells were significantly inhibited, and promoted cell apoptosis. In contrast, if the expression of RAD51-AS1 was silenced, the above anticancer phenomena were restored (Fig. 5a–e). When western blotting was used to analyze RAD51 protein expression in the cells, we observed that after lncRNA RAD51-AS1 was silenced, RAD51 protein expression was immediately disinhibited (Fig. 5f). On the other hand, when the cells were incubated with both corylin and etoposide (VP16) and when the RAD51 protein was overexpressed simultaneously, the inhibition of DNA repair by corylin was attenuated (Fig. 5g, i). This finding showed that the anticancer mechanisms of action of corylin were due to the regulation of RAD51 expression by RAD51-AS1; corylin induced RAD51-AS1 expression, which interfered with the translation of RAD51 mRNA into protein. This action decreased the DNA damage repair capacity of HCC cells, thereby increasing the toxicity of the chemotherapeutic agent toward HCC cells.

### Corylin can inhibit the growth of HCC tumors and increase the in vivo inhibitory effects of a chemotherapeutic drug on tumors

**Fig. 6:**
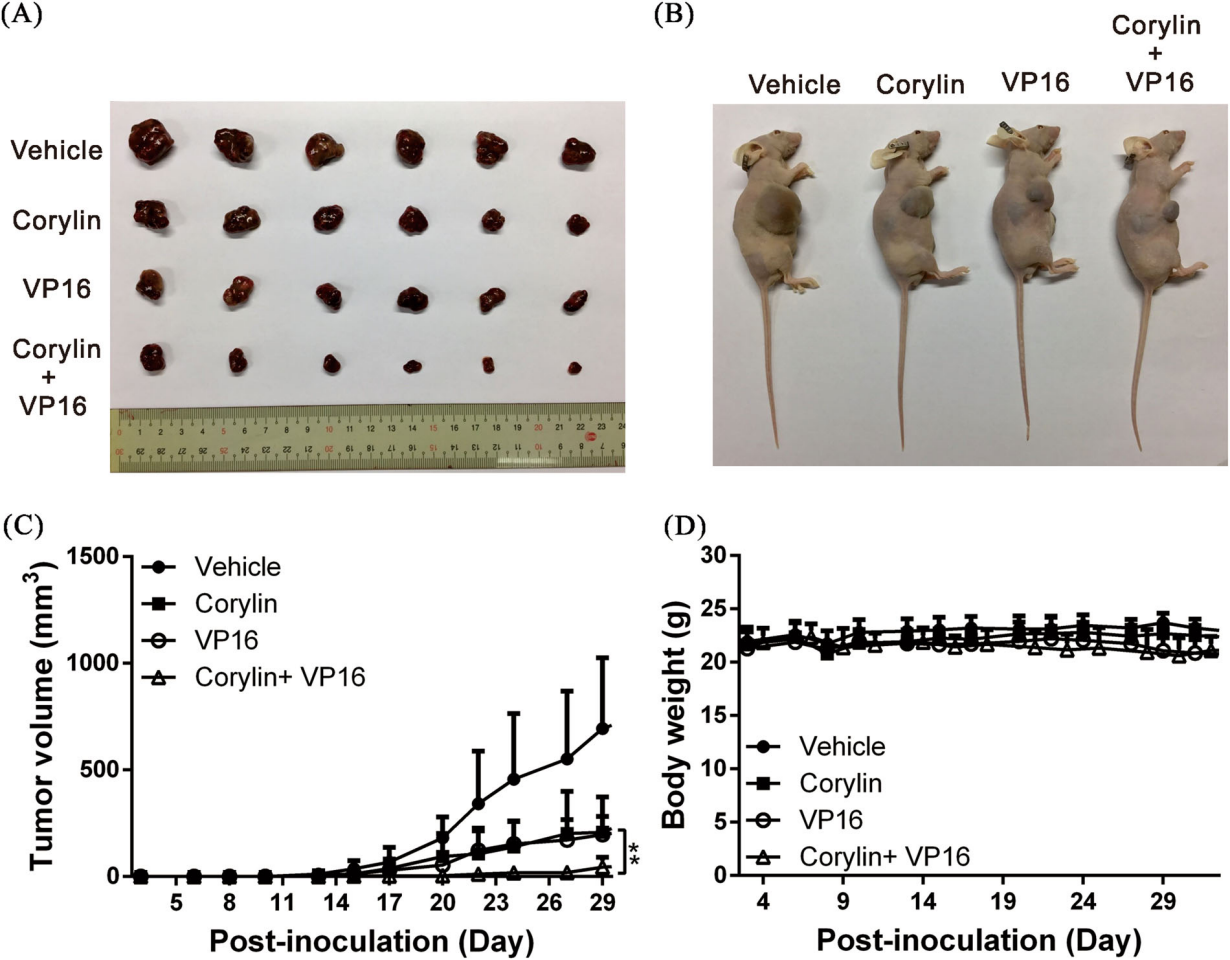
Corylin inhibited the growth of HCC tumors and increased the in vivo inhibitory effects of a chemotherapeutic drug on tumors. **a**, **b** A total of 4 × 10^6^ Huh7 cells were inoculated into nude mice (*n* = 6 each group). The mice with tumors were intraperitoneally (IP) injected 3 days per week with 100 µl of corylin (20 mg/kg of body weight), etoposide (VP16) (5 mg/kg), or an equal volume of dimethyl sulfoxide (DMSO), which served as the control. Representative images show the tumor xenografts at 4 weeks post-implantation. **c** The tumor volumes were calculated every 3 days after injection. The volume of each tumor was calculated as follows: length × width^2^ × 0.5. The cars indicate the S.D. **p < 0.01. Body weights were calculated every 3 days after injection and shown in **d**

To verify the above experimental results, we used a mouse xenograft model to analyze the inhibitory effects of corylin on tumor growth. The results showed that compared with the control group, which was given only vehicle (DMSO), treatment with corylin alone or etoposide (VP16) alone significantly inhibited the growth of mouse tumors. When corylin was administered in combination with etoposide (VP16), the antitumor effects increased by >50% compared with each drug alone (Fig. 6a–c), and this finding was consistent with the results of the in vitro cell experiments. In addition, the corylin treatments did not significantly affect the weights of the mice during the experimental period (Fig. 6d), suggesting that corylin was not toxic to the mice at the dose tested.

**Fig. 7:**
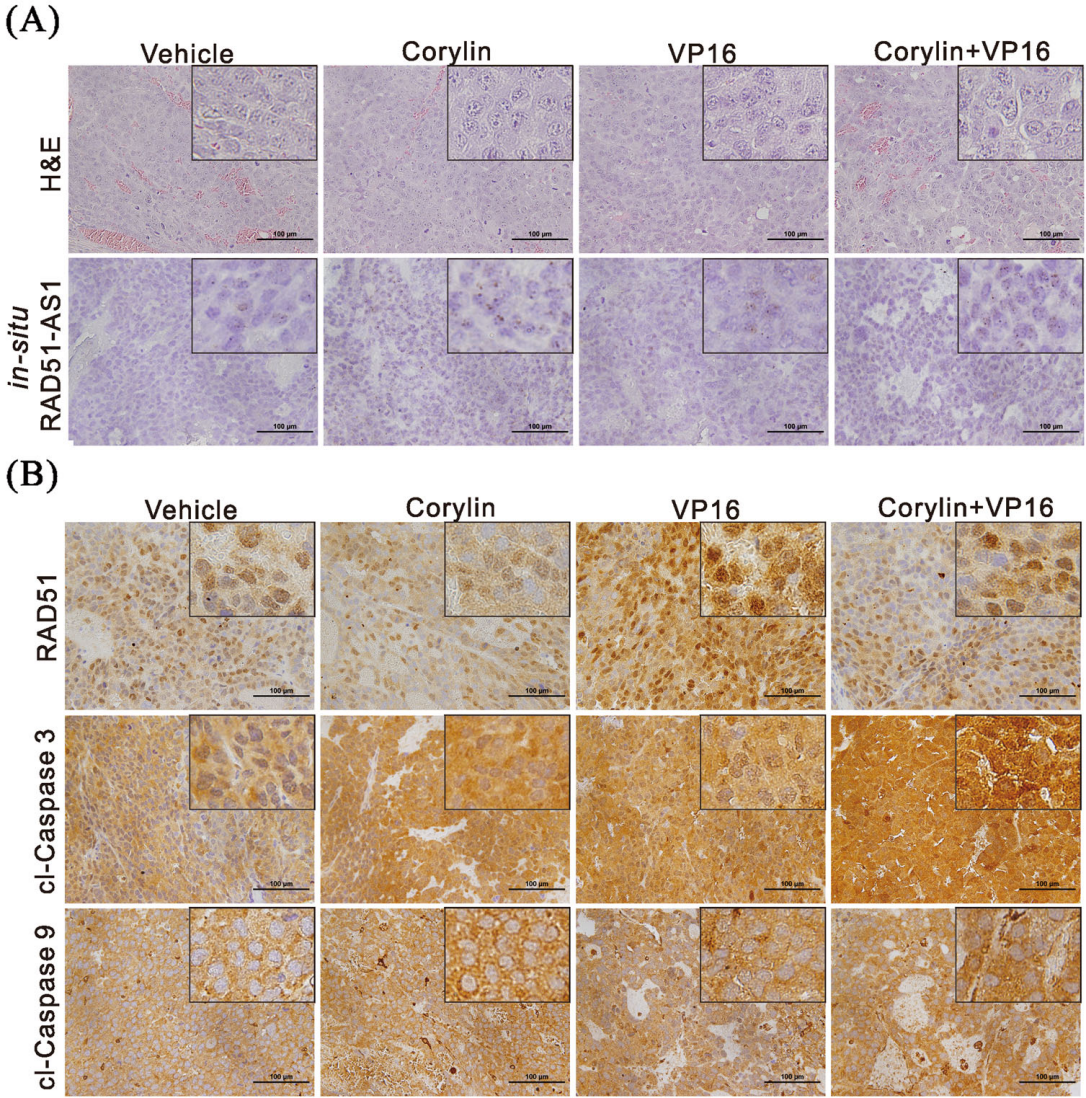
Corylin enhances etoposide (VP16)-induced apoptosis through lncRNA RAD51-AS1-mediated inhibition of DNA damage repair. **a** The xenograft tumors were excised from the mice at the end of the experiment. Histological analysis was performed using H&E staining and in situ hybridization for the detection of RAD51-AS1 level. **b** Immunohistochemical staining represented the effect of corylin and etoposide (VP16) on RAD51, cleaved caspase 3 (cl-caspase 3), and cleaved caspase 9 (cl-caspase 9) protein expression in mice xenograft tumors. Magnification: ×400

We next used in situ hybridization to analyze the expression level of RAD51-AS1 in the mouse tumor tissues and found that RAD51-AS1 expression was significantly higher in the corylin-treated mice than in the control group (Fig. 7a). In addition, we carried out an immunohistochemical staining analysis of the mouse tumor tissues and found that corylin significantly inhibited RAD51 expression in the tissues and upregulated the cleavage of caspases 3 and 9, thus promoting apoptosis (Fig. 7b). The above results showed that corylin could inhibit DNA damage repair in HCC cells by inhibiting RAD51 expression, thereby strengthening the cytotoxic effects of chemotherapy and radiotherapy.

## Discussion

DNA repair is an important mechanism by which cells maintain genomic stability and integrity, and this process is strictly regulated in organisms^43–45^. Nevertheless, the DNA repair systems of many cancer cells are excessively activated due to mutations, resulting in overactive DNA repair. This phenomenon desensitizes cancer cells to DNA damage induced by chemotherapy and radiotherapy and is a mechanism through which cancer cells develop drug resistance^46, 47^. Therefore, in clinical practice, DNA repair inhibitors are administered as adjuvant agents to increase the efficacy of chemotherapy and radiotherapy^48–50^. This study has revealed that corylin that is extracted from *P. corylifolia* L. can inhibit DNA repair in cells and that this effect is mediated by the induction of lncRNA RAD51-AS1. This change then interferes with RAD51 mRNA translation into protein and inhibits HR. To the best of our knowledge, this study is the first to show that corylin can inhibit DNA repair and to report that corylin can regulate lncRNA expression.

This study indicates that in addition to inhibiting DNA repair mechanisms, corylin can inhibit the proliferation, migration, and invasiveness of HCC cells, suggesting that corylin may participate in the regulation of other cancer suppression pathways. Whole-transcriptome sequencing analysis revealed that in addition to RAD51-AS1, corylin can regulate the expression of many lncRNAs. These lncRNAs may be the regulatory factors through which corylin inhibits cancer. Further research is necessary to understand the exact roles and regulatory mechanisms of these lncRNAs, particularly antisense lncRNAs. Our next set of experiments will verify these target genes and their regulatory mechanisms.

Several studies have noted that the majority of antisense lncRNAs can bind to a sense strand (mRNA) to form double-stranded RNA. This structure can stabilize and increase protein expression (e.g., PCNA-AS) or trigger RNA interference mechanisms that cause RNA degradation or interference with protein synthesis, as is the case for RAD51-AS1 in this study^51–55^. How the cell selects one of these regulatory mechanisms remains unclear. We can hypothesize that this mechanism is determined according to the length of the double-stranded RNA or the complementarity region, but this notion requires further study for verification.

Currently, the understanding of the function of lncRNA-RAD51-AS is limited. A recent study reported that RAD51-AS1 can promote cell cycle progression and inhibit apoptosis of ovarian cancer cells, and patients with higher RAD51-AS1 expression have poorer prognosis^56^. Another study indicated that RAD51-AS1 can enhance RAD51-dependent DNA repair capacity in breast cancer cells^57^. However, in this study, we found the opposite results, specifically that RAD51-AS1 can inhibit RAD51 translation and enhance the effectivity of chemotherapy and radiotherapy in liver cancer cells. These studies illustrate that RAD51-AS1 may have diverse regulatory functions in different cells. It is presumed that there may be mutation sites in the RAD51-AS1 sequence or due to the diverse gene background patterns in different cell lines. However, the detailed reason requires further investigation.

At present, the upstream regulatory mechanisms of RAD51-AS1 are not clear, and more studies are needed to clarify how corylin regulates RAD51-AS1 expression. We carried out a promoter prediction analysis of possible transcription factors that could regulate RAD51-AS1 expression and found that transcription factors such as EGR1, p53, and FOXA2 were good candidates. On the other hand, no studies have addressed the regulation of these proteins by corylin. Accordingly, whether corylin regulates the expression of these transcription factors to regulate RAD51-AS1 expression will be the aim of the next phase of our research. In summary, this study shows that corylin can inhibit DNA repair in HCC cells in cell experiments and in an animal model and reveals the corylin mechanisms of action. Corylin holds promise as an adjuvant drug for HCC treatment because this agent can strengthen the therapeutic efficacy of chemotherapy and radiotherapy.

## Materials and methods

### Cell lines, antibodies, corylin, siRNA, and plasmid construction

The HCC cell lines Huh7 and HepG2 were purchased from the American Type Culture Collection (Manassas, VA, USA) and cultured in Dulbecco’s modified Eagle's medium (DMEM; GIBCO, Gaithersburg, MD, USA), containing 10% fetal bovine serum, at 37 °C in a 5% CO_2_-humidified incubator. Polyclonal antibodies against RAD51, *r*H_2_AX, cleaved caspase 3, cleaved caspase 9, and β-actin were purchased from Cell Signaling Technology (Beverly, MA, USA) and Genetex (Irvine, CA, USA). Secondary antibodies were purchased from Santa Cruz Biotechnology (Santa Cruz, CA, USA). The corylin powder (which is characterized by a purity above 98% as measured by high performance liquid chromatography (HPLC)) was purchased from Shanghai BS Bio-Tech Co., Ltd (Shanghai, China). Commercialized si-RAD51-AS1 and negative-control siRNA were purchased from Thermo Fisher Scientific (MA, USA). pCDNA3.1-RAD51-AS1, a CMV-based expression plasmid containing lncRNA-RAD51-AS1, and pCMV-FLAG-RAD51 plasmid were constructed by GenScript Co. (Piscataway, NJ, USA).

### Measurement of the lncRNA-RAD51-AS1 and RAD51 levels using quantitative real-time RT-PCR

Total RNA from Huh7 and HepG2 cells under different treatments was extracted using an RNeasy mini kit (QIAGEN, Gaithersburg, MD, USA) according to the manufacturer’s instructions. Two micrograms of RNA was subjected to reverse transcription (RT). The RT products were subjected to quantitative real-time RT-PCR to detect lncRNA-RAD51-AS1 and RAD51 expression using the TaqMan gene expression kit (Applied Biosystems, Foster City, CA, USA). GAPDH was used as an internal control.

### Transfection and western blotting analysis

Huh7 and HepG2 cells were seeded in six-well plates at a density of 3 × 10^5^ cells/well overnight. The cells were transfected with 1 μg of either the pCDNA3.1-RAD51-AS1 or pCDNA3.1 plasmid using Lipofectamine 3000 (Invitrogen, Carlsbad, CA, USA) according to the manufacturer’s protocol. Four-eight hours after transfection, cells were washed twice with phosphate-buffered saline (PBS) and lysed in 200 μl of RIPA lysis buffer (Thermo Fisher Scientific, MA, USA) containing protease inhibitors. Proteins (50 μg) from the supernatant were separated by sodium dodecyl sulfate-polyacrylamide gel electrophoresis and transferred to nitrocellulose membranes, followed by western blotting analysis to measure the levels of RAD51, cleaved caspase 3 and 9, and β-actin. The immuno-reactive bands were detected using an ECL chemiluminescence kit (NEN Life Science Products, Boston, MA, USA) and developed using X-ray films. The volume of each band was quantified using ImageQuant 5.2 (GE Healthcare, Piscataway, NJ, USA).

### Cell proliferation assay

The cell proliferation capacity was monitored with an xCELLigence real-time cell analyzer (Roche Life Science, Indiana, USA) according to the manufacturer’s instructions.

### Irradiation and colony-forming assay

To evaluate the radiosensitizing effects of corylin, Huh7 cells were seeded on 6-cm dishes at a density of 2000 cells/well. Cells were either untreated or irradiated with a 137-Cs γ-ray source (Atomic Energy of Canada, Ltd, Ontario, Canada) at a dose rate of 2 Gy/min, followed by treated with 30 μM corylin for 24 h. The cells were then cultured in DMEM without corylin for 10 days. The medium was replaced every 3 days. The resultant colonies were subsequently fixed with methanol and stained with 0.1% crystal violet (Sigma–Aldrich, St. Louis, MO, USA). Visible colonies were imaged and quantified manually.

### Cell migration and invasion assay

The migration and invasion abilities of the cells were analyzed using a wound-healing assay and a transwell assay. For the wound-healing assay, treated cells were seeded to six-well plates and cultured to 90% confluence. Cells were scraped with a p200 tip (time 0), and the medium was replaced with low-serum culture medium in the presence or absence of 30 μM corylin. The migration distances of the cells were measured from images (five fields) taken at the indicated time points.

The migration and invasion abilities of cells were assessed using ThinCert Tissue Cell Culture Inserts (Greiner Bio-One, Kremsmunster, Austria) that contained a membrane with a mean pore size of 8 μm. For the migration assay, the cells were trypsinized and suspended in serum-free culture medium (DMEM) with/without 30 μM corylin to a final concentration of 5 × 10^5^ cells/ml. The lower chambers were filled with 500 μl of complete medium (DMEM supplemented with 10% FBS), and 100 μl of the cell suspension was loaded into each upper chamber. The chambers were incubated in a humidified 5% CO_2_ incubator at 37 °C for 24 h. The cells were fixed with 500 μl of methanol for 15 min, and the cells on the inner surfaces of the upper chambers were wiped away using cotton swabs to remove the non-migrating cells. The membrane was washed with 500 μl of PBS and stained with 500 μl of crystal violet for 20 min at room temperature. After the cells were washed with 500 μl of PBS, the stained cells were imaged using ImagePro 6.2 software. Counts were obtained from five random fields at ×100 magnification. For the invasion assay, the membrane was coated with 30 mg/cm^2^ Matrigel (ECM gel, Sigma–Aldrich, St. Louis, MO, USA) to form a matrix barrier. The procedure followed for the invasion assay was the same as the migration assay except that the permeating time for the cells was 48 h.

### Cell apoptosis assay and flow cytometry

For the determination of cell apoptosis, >70% confluent cells were treated with vehicle (DMSO), 30 µM corylin or 200 µM etoposide (VP16) in fresh culture medium. After 48 h, subconfluent cells were trypsinized, washed with 1 × PBS and resuspended at 2 × 10^6^ cells/ml. A total of 1 × 10^6^ cells were fixed with 100% ethanol for 10 min and subjected to apoptosis assays using the Alexa Fluor® 488 Annexin V/Dead Cell Apoptosis Kit (Thermo Fisher Scientific, MA, USA) according to the manufacturer’s instructions. The apoptotic cells were analyzed using a BD FACSCalibur (BD Biosciences, Franklin Lakes, NJ, USA).

### Comet assay

Huh7 cells were seeded to 24-well plates (4 × 10^4^ cells per well), incubated for 24 h, and treated with 200 µM etoposide (VP16) for 1 h. The etoposide (VP16) was then washed out with PBS, and the cells were treated with different concentrations of corylin or DMSO in etoposide (VP16)-free medium for 4 h. The cells were harvested by trypsinization and washed in ice-cold PBS. Then, the cells were mixed with 140 µl of agarose at 43 °C, spread on slides that were precoated with agarose, and solidified for 3 min at 4 °C. The slides were placed in ice-cold lysis solution (2.5 M NaCl, 100 mM EDTA, 10 mM Tris, with 1 ml of Triton X-100 per 100 ml added immediately before use) in the dark for 1 h at 4 °C and then placed in an alkaline electrophoresis solution (0.3 M NaOH, 1 mM EDTA) at 4 °C for 40 min to allow alkaline unwinding. Electrophoresis was performed under alkaline conditions for 20 min at 25 V/cm in a cold room (4 °C). The slides were washed three times with neutralizing buffer (0.4 M Tris-HCl, pH 7.5) and stained with DAPI. Comet images were obtained using a fluorescence microscope (Nikon ECLIPSE Ni-U plus), and the tail moment was calculated using Open comet software.

### In situ hybridization

The expression and localization of lncRNA RAD51-AS1 in mouse tumor tissues were analyzed using the RNAscope 2.0 FFPE Assay-Brown kit with custom-designed probes, according to the manufacturer’s instructions (Advanced Cell Diagnostics, Inc., Hayward, CA, USA).

### Immunofluorescence staining

Immunofluorescence staining was performed as described previously^58^. In brief, Huh7 cells were cultured on chamber slides and treated with 30 µM corylin or 200 µM etoposide (VP16). The slides were fixed with 4% paraformaldehyde in PBS, permeabilized with 0.1% Triton X-100, blocked with 3% bovine serum albumin for 1 h, and incubated for 16 h with antibodies against *r*H_2_AX (Cell Signaling Technology, Beverly, MA, USA). The samples were then incubated with Alexa Fluor 647 donkey anti-goat IgG (A21447, Life Technologies-Molecular Probes). The slides were mounted in Vectashield containing DAPI (4′,6-diamidino-2-phenylindole) (Vector Laboratories, Burlingame, CA, USA), and visualized under a confocal microscope (LSM 700; Carl Zeiss, Jena, Germany).

### HR assay

To determine the percentage of HR, 5 × 10^5^ cells were co-transfected with 1 μg of pDR-GFP (was a gift from Maria Jasin, Addgene plasmid #26475) and I-Sce I (was a gift from Maria Jasin, Addgene plasmid #26477) plasmids with or without corylin treatment for 48 h. Cells were then trypsinized, washed once, and resuspended in PBS. The percentage of GFP-positive cells was quantitated by flow cytometry as previous described.

### Tumor formation assay in a nude mouse model

Six-week-old male BALB/c nude mice (purchased from the National Laboratory Animal Center, Taipei, Taiwan) were maintained under specific pathogen-free conditions and manipulated according to protocols approved by the Institutional Animal Care and Use Committee (IACUC) of Chang Gung Memorial Hospital. A total of 4 × 10^6^ Huh7 cells were resuspended in 100 μl of saline with 50% Matrigel (BD Biosciences), and the suspensions were subcutaneously implanted into the left and right flank regions of the mice. All tumors were grown for 1 week before drug treatment was initiated. At the beginning of the second week, the mice with tumors were intraperitoneally (IP) injected three days per week with 100 µl of corylin (20 mg/kg of body weight), etoposide (VP16) (5 mg/kg) or an equal volume of dimethyl sulfoxide (DMSO), which served as the control. The subcutaneous growth of the tumors was measured every 3 days, and the tumor volumes were calculated using the following equation: length × width^2^ × 0.5. Twenty-four days after drug administration, the mice were sacrificed, and the tumors were subjected to immunohistochemical staining.

### Immunohistochemistry

The tumors of the mice were fixed in formalin and embedded in paraffin. The 2-μm-thick consecutive sections were cut from the paraffin-embedded tissue blocks and floated onto glass slides. The slides were first incubated at 65 °C for 1 h and then deparaffinized in xylene, rehydrated in graded ethanol solutions, and boiled in Trilogy reagent (Cell Marque, Rocklin, CA, USA) for 10 min for antigen retrieval. After washing with 1 × PBS, the slides were immersed in 3% hydrogen peroxide for 10 min to suppress endogenous peroxidase activity. After triple-rinsing with 1 × PBS, the sections were exposed to the appropriate primary antibodies for 1 h at room temperature, after which they were rinsed three times with 1 × PBS and incubated with a biotinylated secondary antibody (Dako, Glostrup, Denmark) for 25 min. After three rinses with 1 × PBS, the slides were treated with horseradish peroxidase-conjugated streptavidin for 25 min. The peroxidase activity was developed with DAB (Dako), followed by counterstaining with hematoxylin.

### Statistical analysis

All data were recorded as continuous variants and analyzed using Student’s *t*-test. All statistical analyses were performed using SPSS 16.0 and Excel 2007. All statistical tests were two-sided, and the *p*-values of significance were established at *<0.05, **<0.01, or ***<0.001.
